# Preliminary evaluation of the safety and efficacy of glucose solution infusion through the hepatic artery on irreversible electroporation focusing

**DOI:** 10.1038/s41598-023-33487-3

**Published:** 2023-05-02

**Authors:** Amirhossein Sarreshtehdari, Fernando Burdio, Borja López-Alonso, Óscar Lucía, José Miguel Burdio, María Villamonte, A. Andaluz, F. García-Arnas, E. Berjano, Xavier Moll

**Affiliations:** 1 Department of Experimental and Health Sciences, Hospital del Mar Medical Research Institute (IMIM), Universitat Pompeu Fabra, Barcelona, Spain; 2 General Surgery Department, Hospital del Mar Medical Research Institute (IMIM), Universitat Pompeu Fabra, Barcelona, Spain; 3 Department of Electronic Engineering and Communications, University of Zaragoza, 50018 Zaragoza, Spain; 4 Hospital del Mar Medical Research Institute (IMIM), Barcelona, Spain; 5 Departament de Medicina i Cirurgia Animals, Facultat de Veterinària, Universitat Autònoma de Barcelona, Barcelona, Spain; 6 BioMIT, Department of Electronic Engineering, Universitat Politècnica de València, Valencia, Spain; 7 Fundació Hospital Clínic Veterinari, Universitat Autònoma de Barcelona, Bellaterra, Spain

**Keywords:** Cancer, Diseases, Health care, Medical research

## Abstract

Due to electrical features of the tissue, such as impedance, which have a significant impact on irreversible electroporation (IRE) function, the administration of glucose solution 5% (GS5%) through the hepatic artery would focus IRE on scattered liver tumors. By creating a differential impedance between healthy and tumor tissue. This study aimed to determine the effects of the GS5% protocol on healthy liver tissue and its safety. 21 male Athymic nude rats Hsd: RH-Foxn1^mu^ were used in the study. Animals were split into two groups. In group 1, a continuous infusion through the gastroduodenal artery of GS5% was performed to measure the impedance with a dose of 0.008 mL/g for 16 min. In group 2, the animals were divided into two subgroups for infusions of GS5%. Group 2.1, at 0.008 mL/g for 16 min. Group 2.2 at 0.03 mL/g for 4 min. Blood samples were collected after anesthesia has been induced. The second sample, after catheterization of the artery, and the third after the GS5% infusion. All the animals were sacrificed to collect histological samples. The survival rate during the experiment was 100%. A considerable impact on the impedance of the tissue was noticed, on average up to 4.31 times more than the baseline, and no side effects were observed after GS5% infusion. In conclusion, impedance alteration by Glucose solution infusion may focus IRE on tumor tissue and decrease IRE’s effects on healthy tissue.

## Introduction

Electroporation (EP) is one of the most garnered interests in surgery and biomedicine. Scientists are trying to develop it since it is a promising non-thermal technique to minimize the invasion degree of ablation surgeries^1–4^. Depending on the desired procedure, EP could be reversible (RE), when the permeabilization in cells is temporal it can be a functional tool in other aspects of therapy, such as gene therapy and electrochemotherapy^5–8^. If the involved electric field is beyond the threshold of cells, they would be unable to shut the pores, thus leading to cell death due to the loss of homeostatic mechanisms and creating permanent instability in the cell membrane. This phenomenon is called irreversible electroporation (IRE)^9^ which can destroy tumor cells by localized non-thermal procedures. Regarding using electrical fields to make changes in the cell membrane permeability, the permeabilization of the cell membrane is exceedingly increased through the disturbance of Na^+^ and K^+^ gradient^10^, it can be a functional tool in other aspects of therapy, such as gene therapy and electrochemotherapy^5–8^. EP can destroy tumor cells by localized non-thermal procedures. Cell death occurs because of a disordered Ca^2+^ signaling system, responsible for producing failed regulation of the energy contribution in cells by depolarization of the cell membrane^11,12^. Damage to membranes may result from EP, which also promotes cell repair. EP damages membrane proteins by generating lipid peroxidation, which results in the emergence of membrane pores. Because several cell death processes overlap, it is still unclear what is the main cause of cell death following electroporation. When the plasma membrane is disrupted, Ca^2+^ ions from the extracellular environment might enter the cell, altering intracellular calcium homeostasis. Since Ca^2+^ is a universal carrier of biological information, EP can activate several cell signaling pathways, such as stress or cell death pathways Even if the resulting pores are sealed again^13^.

In IRE, the electrical field destroys target tissues by inducing short high electric pulses in microseconds widths (80–100 µs)^14,15^. The IRE method does not preserve the surrounding healthy tissue that is very dependent on the Ohmic Characteristic of tissue under the treatment^16–20^. However, it is a non-thermal technique to spare the extracellular matrix but induces apoptosis in healthy and tumor tissue where the electric field applies^21,22^.

Achieving perfect irreversible electroporation requires precision and improving the selectivity of the process^23^ to raise the concentration of the electric field on target cells. Interestingly, hepatic tumor nodules lack sinusoids and are only supplied with blood from the hepatic artery^24–26^. This fact is currently used for identifying tumors by injection of contrast agents with CT or MRI. Therefore, a hypersaline infusion (HI) through the portal vein or a non-electrolyte solution (for instance, glucose solution 5%) through de hepatic artery would change the electrical conductivity of the healthy and tumor tissue of the liver so that, when a potential difference is applied between the opposite sides of the liver, the electrical field magnitude in the tumors would be significantly larger than in the rest of the tissue. Thus, it would be possible to cause electroporation in tumor cells and avoid this phenomenon in healthy liver tissue^27^.

**Figure 1:**
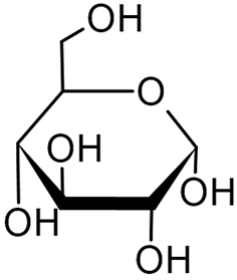
Molecular structures of Glucose. The corresponding solution dissolved in deionized water with concentration of 5%.

In our previous studies, we infused HI through the portal vein to concentrate the electric field in tumor tissue, as we were able to observe conductivity changes of the liver up 1.4 folds higher than tumor tissue^23^, which could focus the treatment on tumor tissue and reduce unwanted damages in the healthy tissue^27^. These results proved that selective electroporation would be available while the electrical field is modified by conductive fluids such as HI through the portal vein^23^. However, utilization of HI in the portal system leads to a significant risk of possible lethal cytolysis and acidosis^28^. For this reason, we propose a new protocol by infusing deionized solution (Glucose) (Fig. 1) into the hepatic artery^29^ to observe changes in the impedance of the tissue. The present study aims to increase the impedance of healthy liver tissue by infusing a low conductivity compound (GS5%) through the hepatic artery and assess its immediate safety.

## Results

### The glucose infusion method is safe

Infusion of glucose solution was uneventful in all the animals with no repercussions on temperature, heart rate, and oxygen saturation during infusion. However, in group 1, four rats were sacrificed because catheterization was impossible, attributable to the small size of their artery’s diameter before the initiation of the infusion. Technically, the survival rate during the experiment was one hundred percent (7 out of 7).

### Analysis of the biochemical parameters of blood samples

Blood analytical results and body temperature in the animals were evaluated for both groups, G2.1, and G2.2, at three stages, post-induction of anesthesia, pre-infusion, and post-infusion of GS5%. All the blood parameter data are shown in Table 1.

**Table 1:** Median ± range of blood test results from 10 rats. Blood samples were extracted from the jugular vein for the post-induction, pre, and post-glucose infusion.

	Post induction		Pre-infusion		Post infusion	
	G2.1	G2.2	G2.1	G2.2	G2.1	G2.2
pH	7.36 (7.25, 7.44)	7.35 (7.32, 7.42)	7.17 (7.16, 7.17)^a^	7.27 (7.12, 7.41)	7.07 (7.05, 7.36)	7.1 (4.04, 7.21)^b^
pCo_2_ (mm)	50.70 (40.40, 64.70)	51.30 (32.30, 55.50)	91.7 (80.1, 92.8)^a^	55.60 (23.8, 83.9)^c^	66.4 (39.1, 98.6)	94 (67.2, 100)^b^
pO_2_ (mm)	162.00 (98, 184)	201 (96, 408)	83.00 (73, 183)	82.50 (70, 211)^a^	76.4 (71.1, 183)	107 (79, 179)
BE (Eq/L)	3.2 (1.8, 4.4)	4 (3, 4.9)	4.80 (3.3, 5)^a^	− 2.00 (− 12.30, 2)^a,c^	− 10.2 (− 12, 6.6)	0.9 (− 4.1, 2)
HCO_3_ (Eq/L)	29.7 (25.8, 30.30)	30.7 (28.3, 31)	33.60 (32.4, 34)^a^	21.00 (17.9, 34.10)	23.00 (17.1, 28.9)^b^	27.3 (20.1, 31.1)
Na^+^ (Eq/L)	133.7 (130.4, 139.55)	136.2 (129, 140)	134 (133.8, 148.6)	143 (131, 148)	139 (102, 180)	122.4 (118, 139)^b^
K^+^ (Eq/L)	3.5 (3.1, 6.8)	4.00 (3.9, 5.1)	3.70 (3.2, 5.4)	6.80 (6.3, 7.2)^a,c^	5.93 (3.7, 8.7)^b^	5.9 (4, 8.3)
Gluczse (mg/dL)	170.00 (144, 182)	156.00 (150.65, 258)	297 (135, 365)	199 (179.5, 291)	410 (79, 569)	700^b,c^
Temperature (°C)	35.80 (34, 35.9)	36.10 (34.9, 37.7)	34.7 (33.9, 35.9)	35 (34.2, 38.4)	34.3 (33.9, 38.6)	36.70 (36.5, 38.1)

^a^Statistical differences from post-induction.

^b^Statistical differences from pre-infusion.

^c^Statistical differences between G2.1 and G2.2.

The mean catheterization length in groups G2.1 and G2.2 were respectively 46.00 ± 24.10 and 41.50 ± 8.89 min. The catheterization process in one of the rats in group G2.1 was quite challenging, and it took 80 min to complete.

### GS5% infusion increases Impedance in the healthy hepatic parenchyma

**Figure 2:**
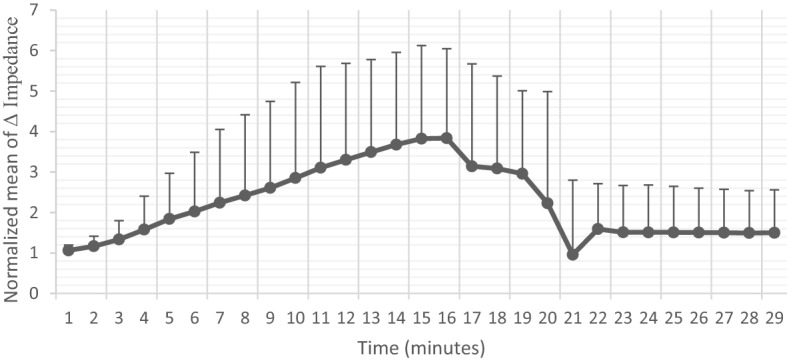
Mean and SD of changes in impedance, after 5% Glucose solution infusion in cases. The graph shows a moderate increase for 16 min more than the outset, then with the end of infusion, a gentle decrease of impedance in hepatic tissue was also perceived. (Normalized mean of Δ impedance: represent the mean of impedance compared to its initial value.). Frequency of the measured impedance is 1000 Hz.

GS5% infusion through the hepatic artery changes healthy tissue impedance. As expected, hepatic tissue impedance was influenced by GS5%. The graph is made by 8 animals, and they are the best cases among sixteen animals, included group G1.1. The mean of maximum hepatic impedance after the infusion for 16 min was up to 4.31 times higher than the mean of minimum impedance baseline. In one of the cases, maximum impedance increased to 6.59 times more than baseline after infusion of GS5% and then, slightly decreased after the termination of infusion and removal of clamps (Fig. 2).

### Histopathology evaluation

At autopsy, macroscopically all the animals showed some degree of pulmonary edema attributable to “postmortem changes”. Four rats have had areas in their lung with alveolar collapse. In all the rats, there are some areas with rupture of the hepatic parenchyma with hemorrhages, and three rats have ruptured capsules. The capsule and parenchyma ruptures were attributable to the manipulation and exteriorization of the liver. The rest of the organs were without significant macroscopic alterations.

**Figure 3:**
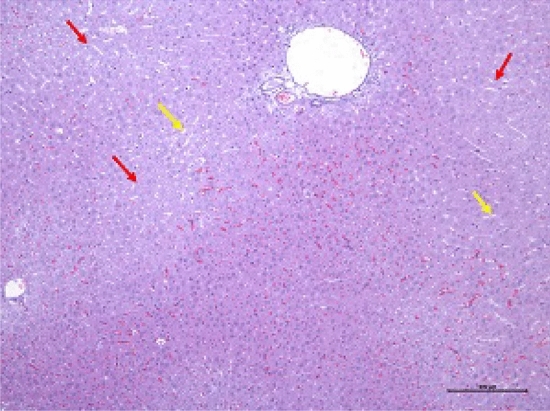
Representative livers after 5% SG protocol at post-infusion. Histological analysis revealed the indemnity of the architecture and morphology of healthy hepatocytes. Paler areas can be seen scattered through the liver parenchyma structure (red arrows). Liver sinusoids can be viewed in the pale areas with almost no erythrocytes (yellow arrows).

In the histopathological study of the liver, all rats had diffused areas of discoloration in their hepatic parenchyma, and it was paler than the other areas. Also, in these areas, there were hepatocytes with cellular degeneration (some without nuclei and others with hyperchromatic nuclei), although they preserved the cellular structure. The hepatic sinusoids in these areas did not have many red blood cells. Obstruction in vessels was infrequently scattered throughout healthy hepatic tissue. No alterations were presented in the cellular component or architecture after the infusion (Fig. 3).

In the lungs, diffuse alveolar edema was observed in all animals. No abnormalities were observed in the spleen, kidney, heart, or brain.

### Stationary simulation of electric field distribution

**Figure 4:**
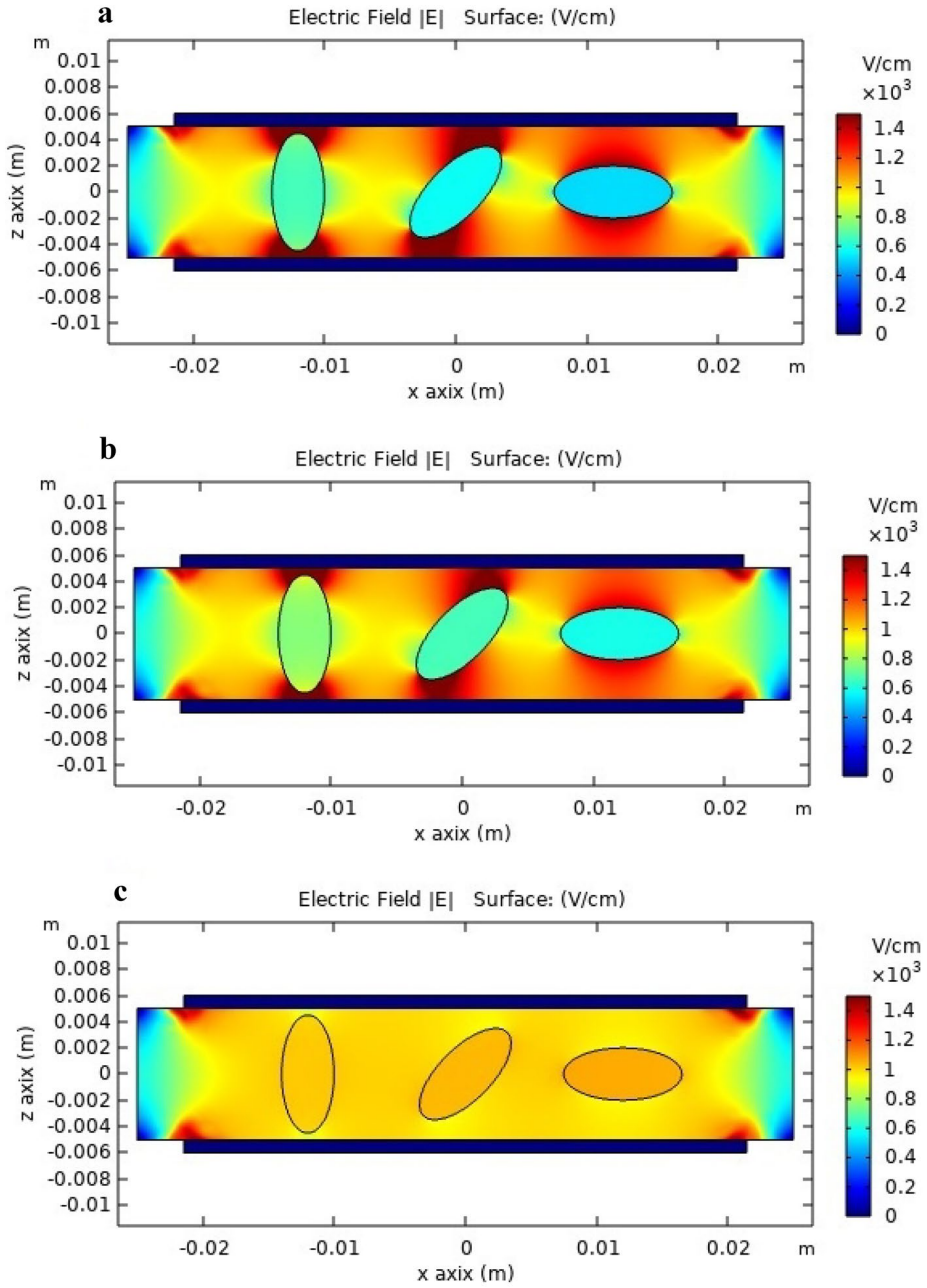
Simulated distribution of the electric field in a 3D FEA model created in COMSOL. The model examines the change in the electric field's distribution in rats' liver tissue with three tumors in three different positions: (**a**) Tumors without serum (tumors that are three times more conductive than healthy tissue), (**b**) Tumors with a lower solution effect (Tumors have 1.7 times less conductive than normal tumor tissue), and (**c**) Tumors with a higher solution effect (Tumors 6.6 times less conductive than normal tumor tissue).

IRE model applied, in 3 tumors in 3 different positions without or with Glucose infusion through the artery at minimum, maximum effects and its corresponding numerical simulation obtained from the received electric field in tumoral tissue. In the first case (without Glucose infusion), there is not selective effect on scattered tumoral nodules. Nevertheless, in Glucose infusion through the Artery, there is an increase on the electric field on scattered tumors (meaning a preferential Electrical field effect on these nodules) because of the increase in healthy tissue conductivity (Fig. 4).

## Discussion

To the best of our knowledge, it is the first study in which an isotonic solution is infused through the arterial inflow to change the electrical conductivity of a specific environment. It could be a rather advantageous situation in the electroporation of liver tumors regarding the lack of sinusoids that tumors display. Both safety and changing conductivity has been demonstrated.

The tissue's physical properties, like impedance, could be a suitable factor to reduce the electrical field throughout the tissues and decrease the damage to the healthy tissues besides the tumor^30^. Following our previous studies on changes in liver tissue conductivity and its safety, we started exploring various biocompatible materials with low toxicity and capable of manipulating tissue impedance to observe the results in the electrical impedance of the tissue^23,27,31^. Finally, GS5% was selected for model^32^. Due to the molecular dissolution of the glucose, glucose does not dissociate into ions in water and hence could not offer the electrical charge. This property, together with the isotonic nature of its solution causes, a considerable impact on the impedance of the tissue with the lowest possible side effects during its liver infusion.

According to the results, we altered electrical impedance in hepatic tissue on an average of up to 4.31 times more than basal impedance. However, individually there was a case with 6.59 times increase in the maximum point. This result could be a prospective for further studies to make changes in the neighborhood environment of hepatic tumors and healthy tissue for allowing the electroporation to distinguish healthy hepatic versus tumoral tissue by differential electrical impedance in future studies. These results are consistent with the results of the simulation of the experiment with the numerical analysis of the data. Contrary to the previous studies in which the infusion of hypersaline caused sharp changes in the relative conductivity of healthy and tumor tissue^23^, the slope of impedance changes in this study is slow both during and at the end of infusion, which can lead to a larger therapeutic window for electroporation. Even though four rats of group 1 were sacrificed before catheterizing and outed from the experiment due to having a too-small hepatic artery, there were no failed cases during the infusion. These results have shown that a 5% glucose solution was a good choice for replacing a hypersaline solution to make a differential electrical environment in the liver tissue with lower risk and higher efficiency.

The infusion method in previous studies was performed through the portal vein^23,28^ due to the presence of various tissues (healthy and tumor). Besides, it was used as a conductive substance to produce higher conductivity in healthy tissue versus tumors. According to many studies, 70% of blood flow in healthy tissue is through the portal vein and 30% through the hepatic artery while the supplied blood in the tumor arrives through the hepatic artery^25,26,29,33^. Therefore, in this study, the hepatic artery was chosen to be engaged to infuse glucose solution. The hypothesis has been that with glucose infusion the impedance changes for both tissues will rise. If there were a tumor, this surge of impedance would be much higher than the healthy tissue and, subsequently, it will create a suitable and long therapeutic window for electroporation. This could be assessed in future studies.

Another discussible finding was blood parameters during the procedure. Mean of post-induction values for each parameter considered as basal concentration in blood (Table 1). Then it was possible to analyze them to see any significant difference between the two stages of pre-infusion and post-infusion with basal values. Statistical analyses have shown there is a difference between blood parameters in post-induction and post-infusion. As expected, hyperglycemia has caused by the large amount of glucose that we administered, especially in group 2.2. The peak of hyperglycemia in animals that are not diabetic does not cause any problems. Lobo showed that a bolus of 1 L of dextrose 5% induces transient hyperglycemia and then, two hours after the infusion, glycemia levels were normalized in healthy people. Moreover, a large number of infused fluids and glucose were voided by urine^34^.

The basal pH in both groups was significantly different from post-infusion. The changes in pH, pCO_2,_ and HCO_3_ observed in our study are associated with anesthesia since the respiratory depression of inhalation anesthesia produces slight hypoxia and hypercapnia, caused to respiratory acidosis^35,36^.

In histopathology results, it has been observed that hepatic congestion occurs mainly due to the large volume of injected fluids (Fig. 3). Although we have not done a long-term study, based on previous experience with the hypersaline solution, which has been prescribed in very high volumes^23,27,28^, we conclude that in the long-term, there will be no problem or alteration in the liver parenchyma. The initial cytolysis observed after hypersaline injection in the earlier study was compensated by rapid liver regeneration at three weeks. Anesthesia could lead to respiratory depression and alveolar collapse. Along with respiratory depression, atelectasis can occur with the administration of oxygen of more than 21%^37–39^. During the liver catheterization of the gastroduodenal artery, a rupture occurred in the liver parenchyma and caused bleeding, which has been described in the histopathology. Regarding these reasons, we believe that alterations are not created by the administration of glucose solution.

Given that no changes in liver structure were observed in the present study, we deem that hepatic congestion resolves or improves over time.

According to the shortcomings of this essay, it counsels future studies to employ larger animals, like rabbits, to avoid complex catheterizing processes due to physiological limitations. Moreover, it would be great to apply the procedure to a tumoral animal model regarding our result. For this reason, our group is working on a tumoral animal model on rabbits to facilitate surgical operation and test this hypothesis. Our trial was limited to examining the status of biochemical factors and blood gases in pre-surgical, pre-infusion, and post-infusion. Long-term monitoring is essential in forthcoming essays to treat possible imbalances caused by infusion, protocol, and anesthesia. These are preliminary results; more reports require to perform.

In conclusion, this study aimed to assess the feasibility and safety of selectively increasing healthy hepatic impedance by infusion of glucose solution through the Hepatic artery as a relief to increase the electric field relatively in scattered tumoral nodules with transhepatic IRE in future studies. Infusion of its solution in the liver could have a considerable impact on the impedance of the tissue. GS5% could be considered a highly valuable solution for decreasing the conductivity of healthy liver tissue for protection against IRE side effects when it is applied to tumor tissue (without portal irrigation) due to its low conductivity and the fact that it is easily eliminated by the kidney and high biocompatibility characters.

Some limitations of the study should also be addressed: (1) this is a preliminary study with a small sample size in the best conditions and we have discarded some cases in which arterial catheterization was impossible; (2) this study has been done in healthy liver. Confirmation of the real benefit into real tumor models should require further experiments.

## Materials and methods

All features of the study had the endorsement of the Ethics Committee on Animal Research of the Government of Catalonia (GE26077/D045906). All the animal experiments in the study had driven following directives 2010/63/ EU of the European Parliament and Council of 22 September 2010, the protection of animals for experimental and scientific approaches. The authors declare that the animal results of the study are reported following ARRIVE guidelines (https://arriveguidelines.org).

### Animal model

The animal model pondered for the study consisted of 21 Athymic nude rats Hsd: RH-Foxn1^mu^ rat (eleven males and ten females), which were eight-week-old at the time of the experiment. The mean and standard deviation of weight in the rats were 456.2 ± 9.32 g and 265.60 ± 14.55 g, males, and females respectively. All the rats were kept under qualified circumstances and alimented with a laboratory animal ratio and water (ad libitum). All animals were monitored for pain, distress, and discomfort recognition in experimental animals by following Morton and Griffiths’ guidelines^40^. Based on our prior studies, animals were split into two groups. In the first group (group 1) (seven males and four females), a continuous infusion through the gastroduodenal artery of GS5% was performed to measure the impedance with a dose of 0.008 mL/g for 16 min, average total volume was 58.6 ± 0.09 mL. In the second group (group 2) (four males and six females), the animals were divided into two subgroups for infusions of GS5%. First, like group one, at 0.008 mL/g for 16 min (group 2.1) and the other at 0.03 mL/g for 4 min (group 2.2). Where the mean of total volume for males and females 54.48 ± 2.4 and 30.8 ± 1.26 mL respectively. Group two were employed to compare biochemical and histopathological alteration before and after SG5% infusion and to investigate the safety in different doses and speeds. (Four times higher and four times faster).

### Blood tests

Three blood tests were collected from animals during the procedure. The first is once after anesthesia has been induced, the second after catheterization of the artery, and the third after the GS5% infusion. Blood sample extraction was performed through the facial vein with a volume of 0.15 mL for each extraction while the temperature was controlled. The electrolytes (Na^+^ and K^+^), Glucose, and blood gases (HCO_3_, BE, pH, pCO_2_, pO_2_) were analyzed immediately with an I-STAT device (Abbott Point of Care Inc, Princeton, NJ, USA).

### Anesthesia, surgery procedure, and infusion

General anesthesia was satisfactorily performed by inhaling isoflurane 4% (IsoVet, BBraun) for induction and 2.5% for maintenance with 100% O_2_ at 0.8 L/min and, subcutaneous analgesia was performed with buprenorphine 0.05 mg/kg (Buprecare, Ecuphar, Spain) and meloxicam 1 mg/kg (Metacam, Boehringer Ingelheim Vetmedica GmBH, Spain). Pulse oximetry, ECG, and temperature were monitored every 5 min during the surgery and infusion procedure using a multi-parameter monitor (VetCare, BBraun, Spain). The abdominal area of each rat was shaved, surgical asepsis routines were properly applied and then, the middle laparotomy was carried out. Due to the placement of sterile gauze in the dorsal region of the rats, after the abdominal incision, the right and left median hepatic lobes were exteriorized. Celiac, hepatic, and gastroduodenal arteries plus the portal vein were accurately identified. Through the gastroduodenal artery, one French catheter (Instech Laboratories, USA) was inserted into the hepatic artery to perform the infusion protocol. The used catheter was fixed at the chosen vessel with non-absorbable Polypropylene Suture 6/0 (Prolene). Surgery length from anesthesia induction until completion of catheterization was controlled. Before the infusion, the celiac artery was clamped with stainless steel micro-vascular clamp (AgnTho's AB, Sweden). The rats were neither intubated nor mechanically ventilated.

**Figure 5:**
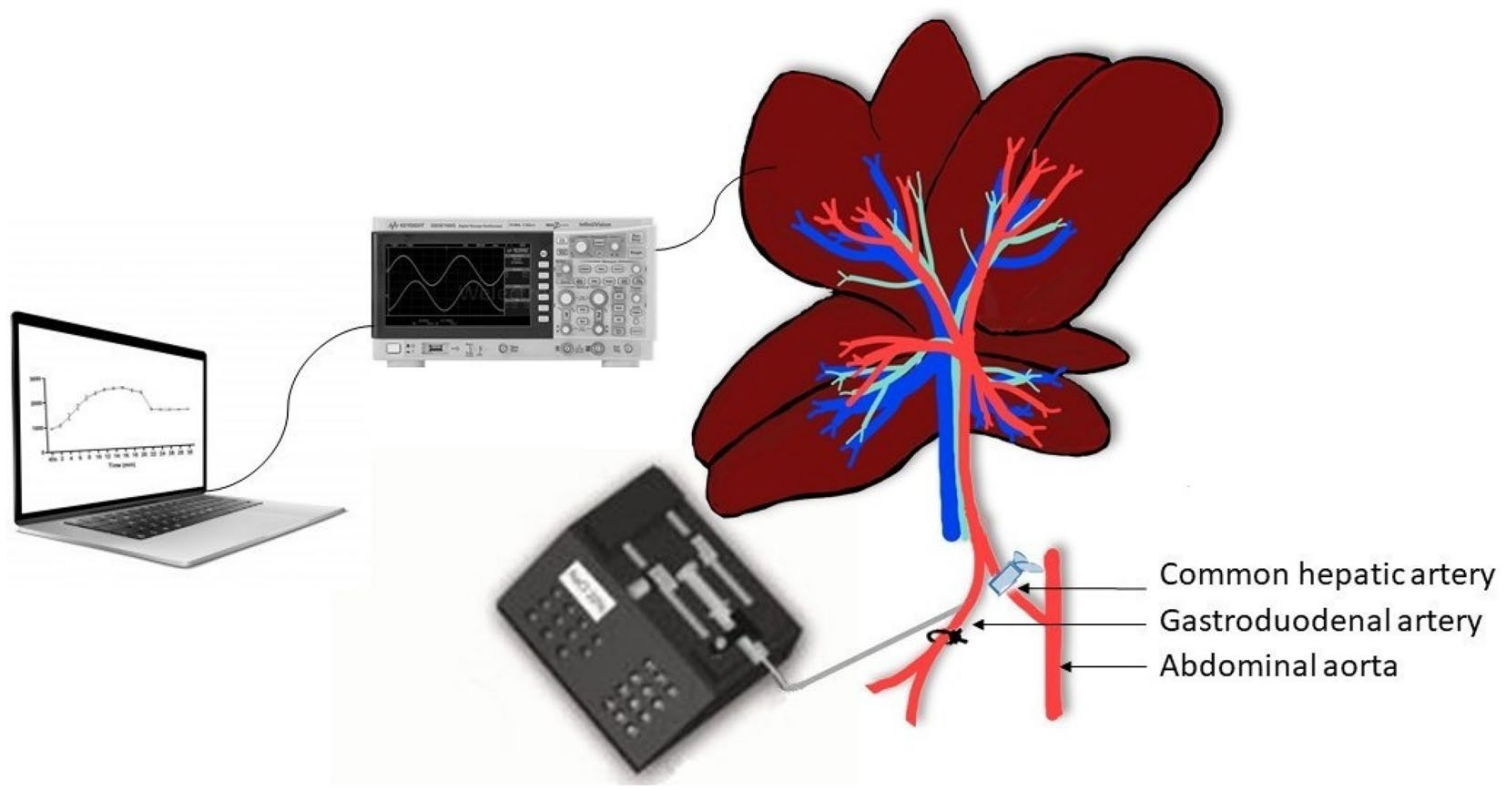
The general setting of the 5% Glucose solution infusion through the hepatic artery in Sprague Dawley rats (A) and in Athymic nude rats. After exposing the spleen at the abdominal midline, GS5% infusion was performed by trans-splenic puncture with a pump. GS5% infusion through the gastroduodenal artery was performed.

Once the gastroduodenal artery was catheterized, the right and left median hepatic lobes were returned to physiological position and the electrodes were placed in the right and left hepatic lobe. Subsequently, the animals were infused with GS5% (Glucosado 5% Braun, BBraun, Spain) using an infusion pump (Fig. 5).

When the infusion protocol was completed, all the rats were sacrificed according to “modified Morton and Griffiths criteria” to assay signs of pain or irritation^40^ by injection of sodium pentobarbital (Dolethal, Vetoquinol, Spain).

### Electrical impedance measurement

**Figure 6:**
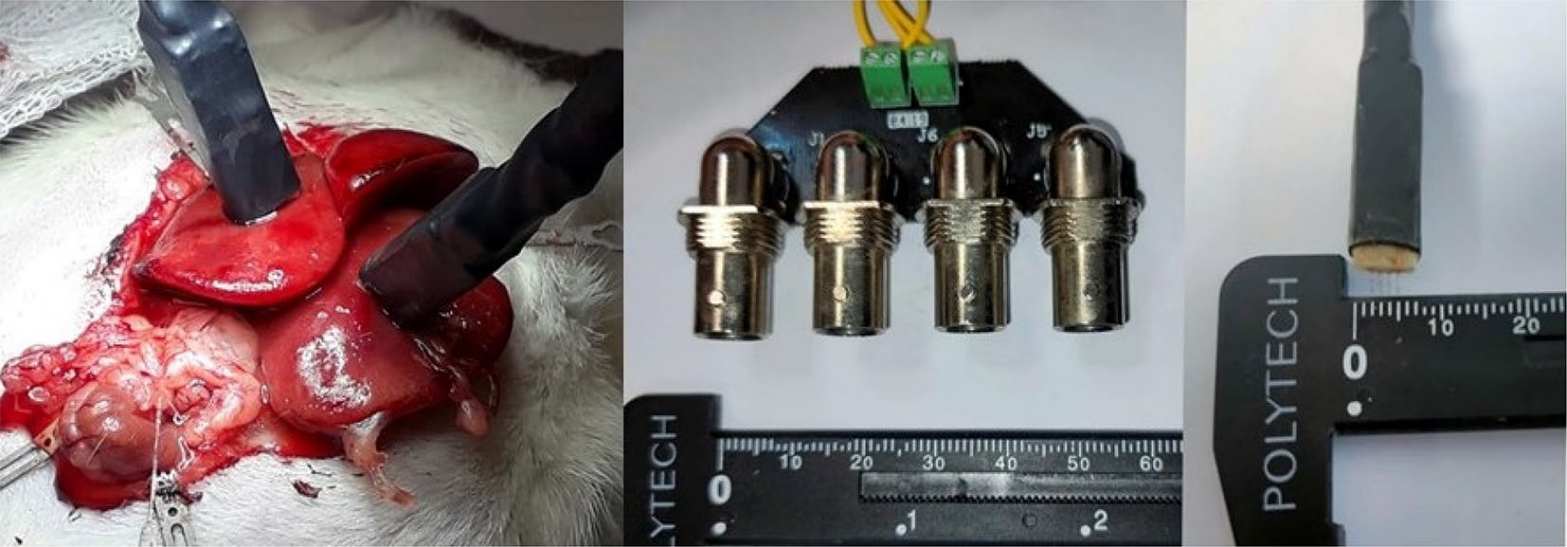
Two tetrapolar setup electrodes measured healthy hepatic impedance in healthy tissue and data were collected with a multifrequency device and gathered in a computer.

Hepatic impedances were accurately measured by using Tetrapolar electrodes with a Kelvin connection, which consisted of four surgical steel needles with a diameter of 0.2 mm, separated by 1 mm and with a penetration depth of 3 mm^27^ (Fig. 6). The standard setup included two Keysight E498A impedance analyzers with a bandwidth between 20 Hz and 300 kHz. The impedance analyzers were monitored by MATLAB software via USB communication. There were two electrodes inserted at the right and left lobe of the liver, with four needle shapes and the most superior stability and completely fixed in the tissue. Electrical impedance was measured at ten excitation frequencies from 1 to 100 kHz at a rate of 0.5 Hz (Fig. 5).

### Histopathological samples

For the histological studies, at necropsy, the brain, lung, heart, liver, spleen, and kidneys were collected from rats. Tissue samples were immediately removed and fixed in formaldehyde and embedded in paraffin. Paraffin sections of tissues were cut into 3 μm thickness and stained with Hematoxylin and Eosin (HEOS)^41^ for light microscopy examination.

### Statistical analyses

All statistical analyses were developed with SPSS statistical software package (SPSS, version 21, IBM, Armonk, NY, USA). Normality was tested using the Shapiro–Wilk statistic. For bio-parameters of blood samples, Mann–Whitney Test and Wilcoxon test were used for non-parametric data to make pairwise comparisons of the impedance of healthy tissue before and after GS5% infusion, in both groups. A P-value of < 0.05 was considered statistically significant.

### The FEA model

COMSOL Multiphysics 5.3a software (COMSOL Inc., Burlington, MA, USA) was used to simulate stationary simulation of electric field distribution in tumor in the FEA model with the injection of 5% solution at the basal impedance of tissue (impedance in tumor tissue is Lower than the healthy tissue)^42–44^. A minimum increase of impedance (1.7 times) and maximum increase of impedance (6.6 times). Applied 500 V to a 0.5 cm thick, so in an ideal situation an average field of 1000 V/cm would be expected^42,44^. Tissue conductivity has been made reliant on the electric field module to improve model accuracy. The parameters they utilized are shown in the Table 2.

**Table 2:** The parameters utilized in 3D modeling of the tissue conductivity based on the electric field module.

	*σ*_*0 *_(s/m)	*σ*_*f *_(s/m)
Liver	0.04	0.12
Tumor	0.2	0.7

*σ*_*0*_ and *σ*_*f*_ are the initial and final electric conductivities of the tissue, respectively^44^.

## Data Availability

The datasets used and analyzed during the current study are available from the corresponding author upon reasonable request. Any restrictions on the availability of materials or information must be disclosed to the editors at the time of submission.
